# Predicting Current Glycated Hemoglobin Levels in Adults From Electronic Health Records: Validation of Multiple Logistic Regression Algorithm

**DOI:** 10.2196/18963

**Published:** 2020-07-03

**Authors:** Zakhriya Alhassan, David Budgen, Riyad Alshammari, Noura Al Moubayed

**Affiliations:** 1 Department of Computer Science Durham University Durham United Kingdom; 2 Computer Science Department College of Computer Science and Engineering University of Jeddah Jeddah Saudi Arabia; 3 College of Public Health and Health Informatics Health Informatics Department King Saud bin Abdulaziz University for Health Sciences Riyadh Saudi Arabia; 4 King Abdullah International Medical Research Center Ministry of the National Guard - Health Affairs Riyadh Saudi Arabia

**Keywords:** glycated hemoglobin, HbA_1c_, prediction, electronic health records, diabetes, differentiated replication, EHR, hemoglobin, logistic regression, medical informatics

## Abstract

**Background:**

Electronic health record (EHR) systems generate large datasets that can significantly enrich the development of medical predictive models. Several attempts have been made to investigate the effect of glycated hemoglobin (HbA_1c_) elevation on the prediction of diabetes onset. However, there is still a need for validation of these models using EHR data collected from different populations.

**Objective:**

The aim of this study is to perform a replication study to validate, evaluate, and identify the strengths and weaknesses of replicating a predictive model that employed multiple logistic regression with EHR data to forecast the levels of HbA_1c_. The original study used data from a population in the United States and this differentiated replication used a population in Saudi Arabia.

**Methods:**

A total of 3 models were developed and compared with the model created in the original study. The models were trained and tested using a larger dataset from Saudi Arabia with 36,378 records. The 10-fold cross-validation approach was used for measuring the performance of the models.

**Results:**

Applying the method employed in the original study achieved an accuracy of 74% to 75% when using the dataset collected from Saudi Arabia, compared with 77% obtained from using the population from the United States. The results also show a different ranking of importance for the predictors between the original study and the replication. The order of importance for the predictors with our population, from the most to the least importance, is age, random blood sugar, estimated glomerular filtration rate, total cholesterol, non–high-density lipoprotein, and body mass index.

**Conclusions:**

This replication study shows that direct use of the models (calculators) created using multiple logistic regression to predict the level of HbA_1c_ may not be appropriate for all populations. This study reveals that the weighting of the predictors needs to be calibrated to the population used. However, the study does confirm that replicating the original study using a different population can help with predicting the levels of HbA_1c_ by using the predictors that are routinely collected and stored in hospital EHR systems.

## Introduction

Diabetes is a growing medical condition worldwide. Globally, the estimated number of diabetic patients in 2017 was 425 million, and it is expected to be more than 629 million by 2045, an increase of more than 48%. The number of people with borderline diabetes is also rapidly increasing. According to the International Diabetes Federation (IDF), there are 352 million people worldwide who are at risk of developing diabetes [1]. The latest estimates indicate that 35.3% of the adults in the United Kingdom and the United States have prediabetes [2].

Type 2 diabetes mellitus (T2DM) is the most common form of diabetes, accounting for 91% to 95% of all cases [3]. T2DM is difficult to diagnose in its early stages because it does not have clear clinical symptoms. As a result of the slow development of its symptoms, it often stays undetected for a long time [4]. The IDF estimates that half of people with diabetes do not know or feel that they are developing diabetes [1].

Hemoglobin is responsible for transporting oxygen throughout the body’s cells and, when joined with the glucose within the blood, it forms glycated hemoglobin (HbA_1c_) [5,6]. The International Expert Committee, with members from the American Diabetes Association (ADA), the European Association for the Study of Diabetes, and the International Diabetes Federation [7,8], recommends the use of the glycated hemoglobin test to identify adults with a high risk of diabetes [9].

An elevation of HbA_1c_ level in the blood can be related to chronic complications and lead to serious health conditions [10]. Patients with HbA_1c_ levels of 5.5% to 6.0% have a substantial risk of developing diabetes, increased by 25% compared with patients with HbA_1c_ levels less than 5.5%. Furthermore, patients with HbA_1c_ levels of more than 6.0% have a 50% chance of developing T2DM over the next 5 years. Those patients are at 20 or more times higher risk than patients who have a level of 5.0% or less [11].

A study by Huang et al [12] showed that patients with HbA_1c_ levels of 5.7% to 6.5% are likely to develop diabetes in 2.49 years. Not only that, but the trend of the HbA_1c_ test has been shown to be an important factor for predicting mortality for patients with T2DM [13]. Furthermore, nondiabetic people with an elevated HbA_1c_ level have an increased risk of cardiovascular disease [9,14]. Hence, studies suggest that patients with and without diabetes with raised levels of HbA_1c_ should be clinically checked and monitored as a preventive intervention to avoid developing T2DM or cardiovascular diseases [14,15].

Many studies have investigated the correlation between HbA_1c_ and clinical variables using statistical and mathematical approaches [16-19]. However, we are not aware of any that have performed replications of the predictive models on different populations. In this paper, we investigate building statistical models that predict the probability of patients having an elevated level of HbA_1c_. We employ comparative statistical models similar to the models used by Wells et al [2] and apply them to a larger electronic health record (EHR) dataset collected from King Abdullah International Medical Research Center (KAIMRC) [20,21] in Saudi Arabia.

The work by Wells et al [2], which we refer to in this paper as the original study, focused on predicting the level of HbA_1c_ for patients who were not previously diagnosed with diabetes or taking diabetes medications. The data were extracted from the EHR database of Wake Forest Baptist Medical Center in the United States. The authors applied a multiple logistic regression model to create a mathematical equation for calculating the level of HbA_1c_ (≥5.7). The predictors used in the equation were chosen from a list of theoretically associated hyperglycemia variables (laboratory measurements, medication categories, diagnosis, vital signs, demographics, family history, and social history variables). After reducing the model’s variables using Harrell’s model approximation method [22] and removing variables that caused collinearity, the final equation associated 8 independent variables with the result of the HbA_1c_ blood test. Restricted cubic splines (RCS) with 3 knots were used for fitting the continuous predictors into the model [2]. The calculator achieved an accuracy of 77%.

The independent replication of empirical studies is widely regarded as being an essential underpinning of the scientific paradigm. Successful replication of a study by other researchers is considered to be an important step in verifying the original findings and helping to determine how widely they apply.

While the vocabulary associated with replication varies across disciplines [23], the terms employed by Lindsay and Ehrenberg [24] appear to be widely used and recognized, so they will be used in this paper. Lindsay and Ehrenberg categorize replication studies as either (1) close replications or (2) differentiated replications.

First, a close replication seeks to repeat the original study in a way that keeps all the “known conditions of the study the same or very similar” [24]. Hence, such a study employs the same forms of measurement, sampling, and analysis as the original, while also seeking to keep the profile of any set of participants as close to the original as possible. A close replication aims to test the hypothesis that, when a given study is repeated under the same experimental conditions as the original study, it should produce the same (or nearly the same) result.

Second, a differentiated replication introduces known variations into what Lindsay and Ehrenberg term “fairly major aspects of the conditions of the study” [24]. Differentiated replications provide a test of how widely the original findings can be generalized, their scope, and the conditions under which they may not hold. For a differentiated replication, therefore, it is expected that some changes in the outcomes are likely to arise, and the question of interest is to what extent and in what form these outcome changes occur.

In an ideal situation, one or more close replications would be used to validate the findings of an original study, followed by a set of differentiated replications used to scope out the extent of their validity by varying different conditions.

For any replication study, it is possible to vary one or more factors from those factors that characterize the way that the study was performed. These may include the team performing the replication, the analysis process, the type of data employed, and the population from which the data were derived. As this study involves analyzing data collected from a human population rather than conducting an experiment or trial, we can expect that using a different team to perform a replication should have no effect. Hence, for a close replication it would be appropriate to use the same analysis tool with EHRs of the same form as used in the original study, but pertaining to a different sample of participants drawn from the same general population used in the original study.

For the differentiated replication reported here, we have used the same form of analysis, but have applied this to a set of EHRs that were derived from a different population. The differences between the forms of the EHRs constituted one difference, but these differences were relatively small. The main difference in the studies arose from the population used. As with the original study, the selection of participants was largely driven by availability. We therefore expected that it was quite possible that there would be some differences in the outcomes, and our main goal was to investigate the extent and form of those differences.

## Methods

### Conduct of the Replication Study

The KAIMRC dataset was collected by the Ministry of National Guard Health Affairs from the EHR systems of National Guard Hospitals in Saudi Arabia for the period from 2016 to the end of 2018. The dataset was then labelled according to the ADA guidelines. Patients with an HbA_1c_ level of 5.7% or more are considered to have an elevated HbA_1c_ and those with lower levels than that are considered normal. The predictors that were selected by the authors of the original study for calculating the level of HbA_1c_, listed in Table 1, were employed in this study, except for race and smoking status. Taking into account that most of the data samples in the KAIMRC dataset are from the same race, the race variable can be omitted, as it has zero variance [25]. Smoking status information is absent from the KAIMRC dataset. However, in the original model used by Wells et al, this was ranked as having the lowest importance of all the predictors. The BMI and non–high-density lipoprotein measures were also absent. However, both can be calculated by using the formulae presented in Multimedia Appendix 1.

**Table 1:** Predictors available in the original study versus King Abdullah International Medical Research Center datasets.

Predictors	Original study dataset	KAIMRC^a^ dataset
Age	√	√
Body mass index	√	√ (calculated)
Estimated glomerular filtration rate	√	√
Random blood sugar (glucose) level	√	√
Non–high density lipoprotein	√	√ (calculated)
Total cholesterol	√	√
Race	√	x
Smoking status	√	x

^a^KAIMRC: King Abdullah International Medical Research Center, Saudi Arabia.

**Figure 1:**
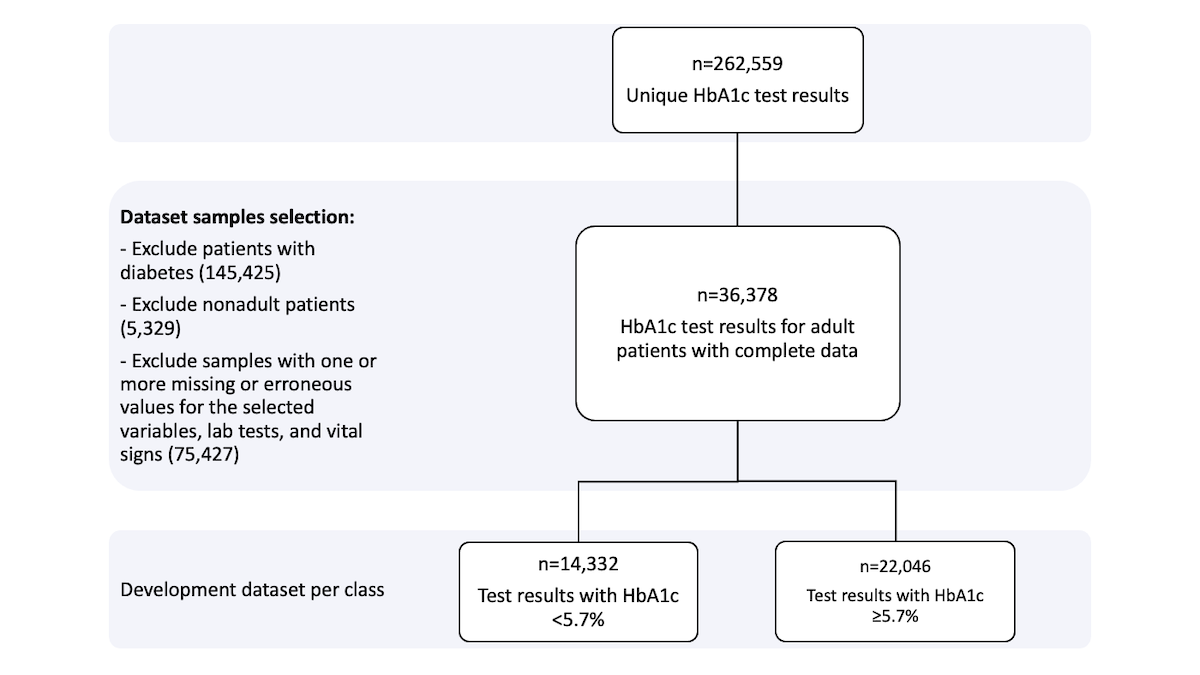
Dataset preprocessing details. HbA_1c_: glycated hemoglobin.

In this study we followed the same sampling approach used in original study. For inpatient visits, only the first day’s data were considered, and in cases of missing values, the first available values for the visit were used. Samples for patients with values of <1% for HbA_1c_ were simply considered to be erroneous readings and were excluded. Similar to the original study, patients diagnosed with diabetes were eliminated from the development dataset (refer to Multimedia Appendix 2 for diabetes diagnostic codes). We avoided intensive interpretation for handling the missing values. Samples with one or more completely missing values were also excluded. This resulted in decreasing the dataset size from the 262,559 samples originally collected to 36,378 samples. Figure 1 shows the detailed preprocessing tasks performed prior to building the statistical models.

The descriptive statistics for the KAIMRC experimental dataset and the dataset used by Wells et al are shown in Table 2. The units used for recording lab tests can differ according to the laboratory guidelines followed by each country. The KAIMRC dataset uses different units than the ones used in the original study for some variables. For instance, the total cholesterol level is measured in milligrams per deciliter (mg/dL) in the original study’s dataset, and in millimoles per liter (mmol/L) in the dataset from the KAIMRC labs. Therefore, the descriptive statistics contain the values using both units. When developing the predictive models, the authors converted the units using the appropriate formulae (see Multimedia Appendix 3). However, the conversion task can be avoided to reduce data preprocessing complexity, as it should not affect the prediction performance for the logistic regression models.

**Table 2:** Descriptive statistics for King Abdullah International Medical Research Center and original study datasets.

Variables^a^		KAIMRC^b^ dataset			Original study^c^ dataset	
		HbA_1c_^d^ <5.7% (n=14,332)	HbA_1c_ ≥5.7% (n=22,046)	*P* value	HbA_1c_ <5.7% (n=16,743)	HbA_1c_ ≥5.7% (n=5892)
Age (years), mean (SD)		45.5 (17.01)	60.5 (14.13)	<.001	48.1 (15.4)	54.8 (14.0)
BMI (kg/m^2^), mean (SD)		29.61 (10.74)	31.50 (12.13)	<.001	30.1 (7.44)	33.0 (8.41)
eGFR^e^ (mL/min/1.73 m^2^), mean (SD)		93.40 (35.19)	82.02 (28.86)	<.001	92.0 (33.0)	87.9 (30.8)
**RBS^f^**				<.001		
	RBS (mmol/L), mean (SD)	5.47 (1.28)	8.30 (4.30)		4.9 (0.7)	5.3 (0.9)
	RBS (mg/dL), mean (SD)	98.5 (23.00)	149.4 (77.47)		88.4 (12.7)	96.1 (16.0)
**Cholesterol**				<.001		
	Cholesterol (mmol/L), mean (SD)	4.59 (1.19)	4.17 (1.16)		4.80 (1.01)	4.96 (1.11)
	Cholesterol (mg/dL), mean (SD)	177.49 (46.01)	161.25 (44.85)		186 (39.4)	192 (43.1)
**Non-HDL^g^**				<.001		
	Non-HDL (mmol/L), mean (SD)	2.85 (1.06)	2.49 (0.99)		3.49 (0.96)	3.72 (1.07)
	Non-HDL (mg/dL), mean (SD)	110.2 (40.99)	96.28 (38.28)		135 (37.4)	144 (41.7)

^a^Refer to Multimedia Appendix 3 for unit conversion formulae.

^b^KAIMRC: King Abdullah International Medical Research Center, Saudi Arabia.

^c^Wake Forest Baptist Medical Center, North Carolina, United States.

^d^HbA_1c_: glycated hemoglobin.

^e^eGFR: estimated glomerular filtration rate.

^f^RBS: random blood sugar.

^g^HDL: high-density lipoproteins.

### Study Design

A complete validation of Wells et al’s calculator using our dataset was not possible due to the absence of the smoking status variable. To validate the approach used in the original study, 3 predictive models (PMs) were built, trained, and tested using the KAIMRC dataset. All models employ multiple logistic regression to create the calculator by associating the chosen and available predictors. After discussion with the authors of the original study, we structured the models as PM1, PM2, and PM3.

PM1 was designed to be as close as possible to the original study’s model. It uses the predictors chosen in the original study: age, BMI, random blood sugar (RBS), non–high-density lipoprotein (non-HDL), cholesterol, and estimated glomerular filtration rate (eGFR). The continuous predictors are fitted to the model using RCS with 3 knots.

PM2 was designed using the same predictors used in PM1 but without RCS fitting.

PM3 was designed after excluding the predictors with the least importance in PM1 and PM2, using a reduced number of predictors and fitted using RCS with 5 knots. The choice of the number of knots for this model was determined by using Stone’s recommendation [26].

The 3 models were validated using the 10-fold cross-validation approach. The measure used to evaluate and compare the results with the original study was the concordance statistic, which is equal to area under the receiver operating characteristic (AUR ROC) curve [27]. To assist with future comparisons, we report measures commonly used for medical research, such as precision, recall, and F1, in the model evaluation. The data preparations are undertaken using Python (version 3.7; Python Software Foundation). The model building and the analysis are carried out in R (version 3.6.0; The R Foundation) using the regression modeling strategies package.

## Results

The development data subset size used for training, testing, and validating the models after data preprocessing was 36,378 samples. Most medical datasets are imbalanced with a majority normal population [28], but 60.60% (22,046/36,378) of KAIMRC dataset patients were found to have elevated levels of HbA_1c_ (≥5.7%), and 39.40% (14,332/36,378) of patients had a normal HbA_1c_ level (<5.7%).

**Figure 2:**
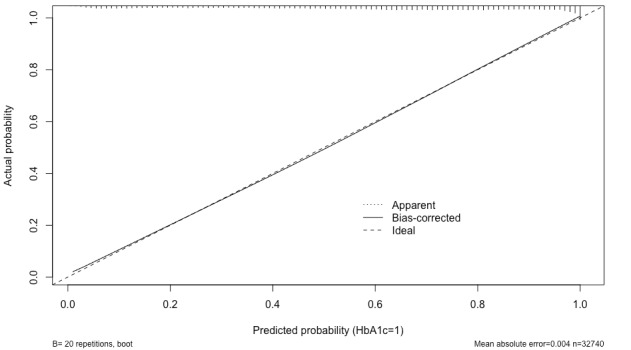
The calibration curve for PM1. HbA_1c_: glycated hemoglobin. PM: predictive model.

**Figure 3:**
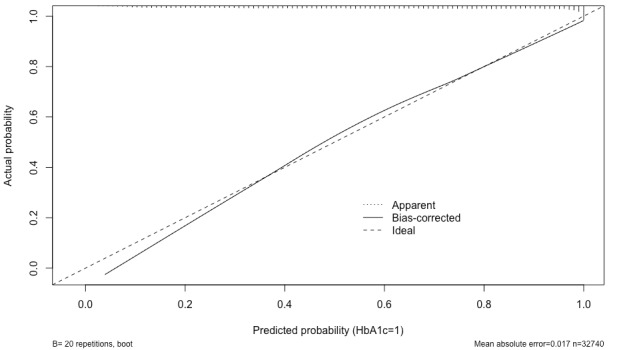
The calibration curve for PM2. HbA_1c_: glycated hemoglobin. PM: predictive model.

Details of the 3 models (PM1, PM2, and PM3) used for the purpose of validating and evaluating the original study are shown in Table 3. This study explores multiple logistic regression models using different numbers of variables, with and without RCS, and with different numbers of knots. PM1 (using a complete set of variables fitted using RCS) achieves an average accuracy of 73.67% and 95% CI of 74% to 77% with a well-calibrated curve. A similar model (PM2), but not fitted using RCS, shows improved accuracy, with an average accuracy of 74.04% and the same 95% CI of 74% to 77%. However, the calibration curve shows better calibration when applying RCS into the models, as shown in Figures 2 and 3.

**Table 3:** Performance of models for glycated hemoglobin elevation prediction.

Model	Variables used	Number of RCS^a^ knots	AUR ROC^b^	95% CI	Recall	Precision	F1
PM^c^1	Complete^d^	3	73.67	74.71-77.51	85.24	77.58	81.23
PM2	Complete	N/A^e^	74.04	74.35-77.16	82.18	78.76	80.43
PM3	Reduced^f^	5	74.73	75.38-78.15	84.40	78.80	81.50

^a^RCS: restricted cubic splines.

^b^AUR ROC: area under the receiver operating characteristic.

^c^PM: predictive model.

^d^All variables (age, random blood sugar, cholesterol, non–high-density lipoproteins, estimated glomerular filtration rate, and BMI).

^e^N/A: not applicable.

^f^Reduced variables (age, random blood sugar, cholesterol, non–high-density lipoproteins, and estimated glomerular filtration rate).

**Figure 4:**
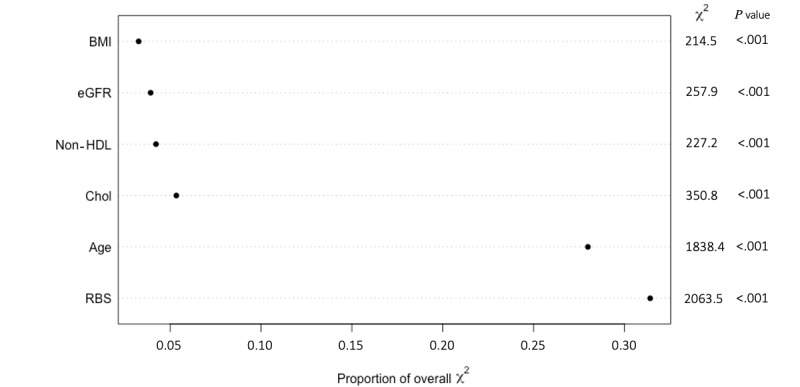
Order of importance of predictors for PM1. Chol: cholesterol. eGFR: estimated glomerular filtration rate. HDL: high-density lipoproteins. PM: predictive model. RBS: random blood sugar.

Figure 4 shows the ranking of importance for the variables used in the PM1 model. PM1 shows a different order of importance for the predictors than the order obtained from the original study. Age and RBS are of great importance in both studies. However, BMI is of the lowest importance when using the KAIMRC population, whereas in the original study it was ranked second.

**Figure 5:**
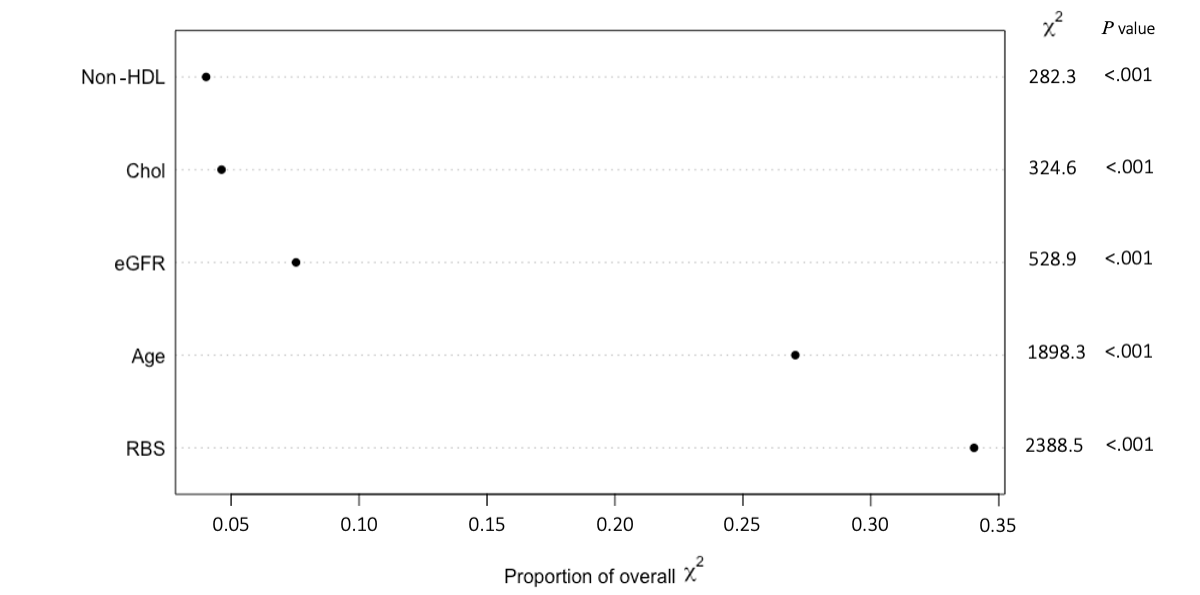
Order of importance of predictors for PM3. Chol: cholesterol. eGFR: estimated glomerular filtration rate. HDL: high-density lipoproteins. PM: predictive model. RBS: random blood sugar.

The PM3 model excludes the variable that showed the lowest importance, BMI. This model, when fitted using RCS with 5 knots, shows better performance using only the 5 predictors (age, RBS, cholesterol, eGFR, and non-HDL). The eGFR shows greater importance when fitted using RCS with 5 knots (>0.05) than when fitted with 3 knots (<0.05). The predictors’ importance order for PM3 is shown in Figure 5. PM3 achieves an average accuracy of 74.73%, with a better confidence interval (95% CI 75%-78%). The calibration curve for PM3 is identical to that of PM1.

When using the PM2 model, the results show agreement with the results from PM1 for 93.27% (33,929/36,378) of predictions. The PM3 model with fewer predictors achieves a better performance and a similar percentage of predictions that are in agreement with the output from PM1 (33,937/36,378, 93.29%). Furthermore, the results show a strong degree of correlation among the probability outputs produced by the 3 models (*r*=0.97).

## Discussion

### Principal Results

Applying the method employed in the original study achieved an accuracy of 73% to 74% using a dataset collected from the Middle East, compared with 77% obtained from using a population from the United States in the original study. The findings from this replication study therefore confirm the conclusion from the original study that this form of modeling can help with predicting the levels of HbA_1c_ in a blood test for nondiabetic patients using predictors extracted from EHR systems.

The order of importance obtained for the predictors used by the multiple logistic regression on our dataset is different from the order of importance produced in the original study. The order for the predictors using the KAIMRC dataset, from the most to the least importance, is RBS, age, eGFR, cholesterol, non-HDL, and BMI. Table 4 shows the importance rankings for the predictors obtained from the original study, as well as the rankings obtained from the 3 models used in this study.

**Table 4:** Predictors importance rankings.

Study		1st	2nd	3rd	4th	5th	6th	7th	8th
Original study		Age	BMI	RBS^a^	Race	Non-HDL^b^	Cholesterol	eGFR^c^	Smoking status
**Replication study**									
	PM^d^1	RBS	Age	Cholesterol	Non-HDL	eGFR	BMI	N/A^e^	N/A
	PM2	Age	RBS	Cholesterol	Non-HDL	BMI	eGFR	N/A	N/A
	PM3	RBS	Age	eGFR	Cholesterol	Non-HDL	BMI (excluded)	N/A	N/A

^a^RBS: random blood sugar.

^b^HDL: high-density lipoproteins.

^c^eGFR: estimated glomerular filtration rate.

^d^PM: predictive model.

^e^N/A: not applicable.

**Figure 6:**
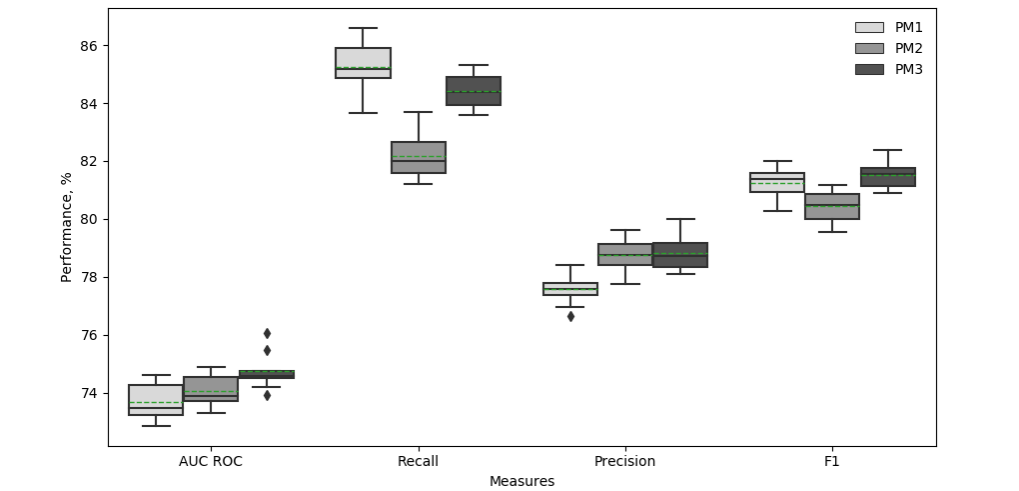
Box plots of the reported measures for the models. AUC ROC: area under the receiver operating characteristic. PM: predictive model.

BMI was one of the most important predictors in the population from the United States and demonstrated higher impact than the RBS and eGFR. However, it shows little importance for predicting the elevation level of HbA_1c_ in the KAIMRC population. Indeed, the simpler calculator with a reduced number of variables (after excluding BMI) is able to achieve better prediction abilities (refer to Multimedia Appendix 4 for details of the calculator). Figure 6 summarizes the 10-folds performance achieved using the reported measures for all models, and reveals that there is a consistent prediction trend for PM3, especially in the AUR ROC, which shows little variation between the folds.

This replication study shows that the ranking of the variables is largely based on the dataset and the model used for prediction. Variables with low importance in the prediction of HbA_1c_ in one population may show greater or lesser importance when the model is applied on populations from different regions of the world. Interestingly, this can also happen when employing different predictive models and with different hyperparameters using the same population (for instance, eGFR shows higher importance when fitted to the model using RCS with 5 knots in PM3 than with 3 knots in PM1 and without RCS in PM2, as interpreted in Table 4).

### Limitations and Future Work

We performed a differentiated replication using a population from a different region that was available to us. The 2 datasets have similar means and standard deviations for most of the variables, such as age, cholesterol, and non-HDL, as described in Table 2. However, there is a significant difference in the body mass index and random blood sugar variables, and the dispersion is large for both variables.

The sample size and class balance affect the learning behavior of the models [29]. The KAIMRC dataset is larger than the one used in the original study by 38%. The class balance is also different, with 26% of patients having elevated HbA_1c_ (≥5.7%) and 74% with normal HbA_1c_ (<5.7%) in the original study compared with 60.60% (22,046/36,378) with elevated HbA_1c_ (≥5.7%) and 39.40% (14,332/36,378) with normal HbA_1c_ (<5.7) in KAIMRC dataset.

Although the population represented in this study is less heterogeneous with regard to ethic groups, the size of the KAIMRC dataset is larger than the one used in the original study. The prevalence of diabetes is also larger, being a sample from the population of Saudi Arabia. In terms of prevalence of diabetes, Saudi Arabia was ranked by the World Health Organization as being the second highest in the Middle East and seventh highest in the world [30], with an 18.3% diabetes prevalence rate, according to the IDF, compared with 10.5% in the United States [31].

In the original study, the model performance was compared with the models developed by Baan et al [32] and Griffin et al [33], which used different datasets [34,35]. The main limitation in the comparison between the original study and the studies by Baan et al and Griffin et al is the absence of some variables that were used to create the calculators (refer to Multimedia Appendix 5 for details about the variables used in the corresponding studies). The same situation applies to this study, as the smoking status variable is missing in the KAIMRC dataset. The smoking prevalence in Saudi Arabia is between 2.4% to 52.3% among different age groups [36]. However, other missing predictors, such as genetic or lifestyle characteristics [37], which are difficult to collect and incorporate into the EHR systems, may help to explain the high rate of elevated levels of HbA_1c_ in the KAIMRC population.

**Figure 7:**
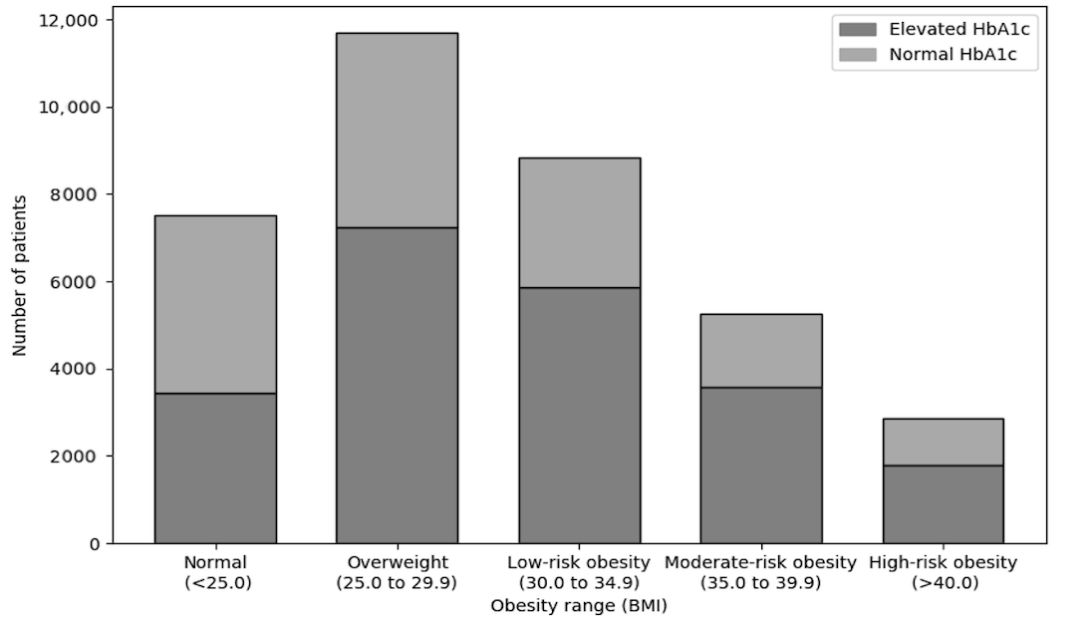
HbA_1c_ elevation for BMI ranges of King Abdullah International Medical Research Center patients. HbA_1c_: glycated hemoglobin.

After eliminating the variables that do not show significant impact on the prediction of HbA_1c_ in the KAIMRC population, the results indicate that different regions in the world can have different weightings of predictors for HbA_1c_ when using the approach of Wells et al. Although there are many studies that have demonstrated the relationship between diabetes prevalence and BMI [38], some studies have shown that the obesity prevalence in Asian countries does not relate to the diabetes prevalence. The risk of diabetes occurs in patients with a lower BMI in Asian countries compared with patients from European countries [39]. The prevalence of obesity in Asian countries is substantially less than in the United States, but Asian countries have a similar or higher prevalence of diabetes [40]. However, neither Yoon et al [39] nor Hu [40] identifies a relationship between nondiabetic patients with elevated levels of HbA_1c_ and obesity. Figure 7 visualizes the class distribution for the BMI variable for the KAIMRC dataset. The figure shows that elevation of HbA_1c_ exists with similar rates between low and high obesity ranges.

Advanced data mining techniques, such as deep machine learning models, are capable of finding hidden and complex correlations in large input spaces and datasets [41]. Recently, machine learning models have shown great success in many domains (eg, natural language processing, image segmentation, and object detection), but there is still a lack of studies that apply those models to the medical domain using EHR data [42]. As stated in the original study, maintaining security and privacy for medical datasets is a challenging task. However, with advanced technologies in data privacy and protection, such as differential privacy and data anonymization techniques [43], it should be possible to minimize the security risk.

### Conclusions

Replication studies provide an invaluable contribution to the validation, generalization, and continuation of scientific research. The differentiated replication presented in this study is aimed at validating the calculator used for predicting HbA_1c_ and evaluating the method used to create the mathematical equation by training the multiple logistic regression algorithm using EHR datasets. The evaluation was performed using a dataset collected from a different population. The original and replicated calculators employ associated predictors that are routinely collected and stored in hospital systems.

As explained in the “Introduction” section, this differentiated replication study used the same method to analyze a different population sample, with some differences in the form of the EHRs. As a replication, it was intended to investigate what changed and did not change in the outcomes.

What did not change appreciably was the accuracy of the results produced using this method, with an accuracy range of 73.6% to 74.7% in our study compared with 77% in the original study. The set of predictors (when these could be compared) also did not change. Thus, given that a close replication of the original study is unavailable, the differentiated replication does confirm that, despite the notable differences between the two datasets, the use of multiple logistic regression is able to provide good predictions of HbA_1c_ elevation levels.

What did change was the order of importance for the set of predictors used in the calculator. Thus, we can conclude that the use of multiple logistic regression for prediction does need to be tuned to the characteristics of the population being assessed. While we cannot wholly rule out the cause of this difference in importance being due to differences in the form of the EHRs, it seems more likely that the characteristics of the population were an important factor.

In terms of the role of replication itself, we would argue that this study demonstrates that while there is little difference in prediction accuracy when using multiple logistic regression with different populations (as might be expected), the influence of the different elements in the set of predictors is different. Due to that, we would argue that the generalization of simple statistical predictive models (calculators) is inappropriate. We suggest that creating advanced predictive models that can learn complex relationships using large multidimensional datasets may be a better way to exploit the increasing volumes of EHR data becoming available. Hence, further work will investigate applying advanced machine learning techniques to predict the elevation of HbA_1c_ using the KAIMRC dataset.

## Appendix

Multimedia Appendix 1 Formulae for the calculated variables.

Multimedia Appendix 2 Lab test and diagnostic codes.

Multimedia Appendix 3 Units conversion formulae.

Multimedia Appendix 4 PM3 Calculator details.

Multimedia Appendix 5 Variables used in the studies.

## Abbreviations

ADA: American Diabetes Association
AUR ROC: area under the receiver operating characteristic
eGFR: estimated glomerular filtration rate
EHR: electronic health record
HbA_1c_: glycated hemoglobin
HDL: high-density lipoprotein
IDF: International Diabetes Federation
KAIMRC: King Abdullah International Medical Research Center
PM: predictive model
RBS: random blood sugar
RCS: restricted cubic splines
T2DM: type 2 diabetes mellitus

## References

[1] Cho N, Shaw J, Karuranga S, Huang Y, da Rocha Fernandes J, Ohlrogge A, Malanda B (2018). IDF Diabetes Atlas: Global estimates of diabetes prevalence for 2017 and projections for 2045. Diabetes Res Clin Pract.

[2] Wells BJ, Lenoir KM, Diaz-Garelli J, Futrell W, Lockerman E, Pantalone KM, Kattan MW (2018). Predicting Current Glycated Hemoglobin Values in Adults: Development of an Algorithm From the Electronic Health Record. JMIR Med Inform.

[3] Ogurtsova K, da Rocha Fernandes J, Huang Y, Linnenkamp U, Guariguata L, Cho N, Cavan D, Shaw J, Makaroff L (2017). IDF Diabetes Atlas: Global estimates for the prevalence of diabetes for 2015 and 2040. Diabetes Res Clin Pract.

[4] Beagley J, Guariguata L, Weil C, Motala AA (2014). Global estimates of undiagnosed diabetes in adults. Diabetes Research and Clinical Practice.

[5] Peterson K P, Pavlovich J G, Goldstein D, Little R, England J, Peterson C M (1998). What is hemoglobin A1c? An analysis of glycated hemoglobins by electrospray ionization mass spectrometry. Clin Chem.

[6] Koenig RJ, Peterson CM, Jones RL, Saudek C, Lehrman M, Cerami A (1976). Correlation of Glucose Regulation and Hemoglobin A in Diabetes Mellitus. N Engl J Med.

[7] International Expert Committee T (2009). International Expert Committee report on the role of the A1C assay in the diagnosis of diabetes. Diabetes Care.

[8] American Diabetes Association (2010). Diagnosis and classification of diabetes mellitus. Diabetes Care.

[9] Ackermann RT, Cheng YJ, Williamson DF, Gregg EW (2011). Identifying Adults at High Risk for Diabetes and Cardiovascular Disease Using Hemoglobin A1c. American Journal of Preventive Medicine.

[10] Bonora E, Tuomilehto J (2011). The pros and cons of diagnosing diabetes with A1C. Diabetes Care.

[11] Zhang X, Gregg EW, Williamson DF, Barker LE, Thomas W, McKeever Bullard K, Imperatore G, Williams DE, Albright AL (2011). Response to Comment on: Zhang et al. A1C Level and Future Risk of Diabetes: A Systematic Review. Diabetes Care 2010;33:1665-1673. Diabetes Care.

[12] Huang C, Iqbal U, Nguyen P, Chen Z, Clinciu DL, Hsu YE, Hsu C, Jian W (2014). Using hemoglobin A1C as a predicting model for time interval from pre-diabetes progressing to diabetes. PLoS One.

[13] Ma W, Li H, Pei D, Hsia T, Lu K, Tsai L, Wei J, Su C (2012). Variability in hemoglobin A1c predicts all-cause mortality in patients with type 2 diabetes. J Diabetes Complications.

[14] Khaw K, Wareham N, Bingham S, Luben R, Welch A, Day N (2004). Association of hemoglobin A1c with cardiovascular disease and mortality in adults: the European prospective investigation into cancer in Norfolk. Ann Intern Med.

[15] Pradhan AD, Rifai N, Buring JE, Ridker PM (2007). Hemoglobin A1c predicts diabetes but not cardiovascular disease in nondiabetic women. Am J Med.

[16] McCarter RJ, Hempe JM, Chalew SA (2006). Mean blood glucose and biological variation have greater influence on HbA1c levels than glucose instability: an analysis of data from the Diabetes Control and Complications Trial. Diabetes Care.

[17] Nathan DM, Kuenen J, Borg R, Zheng H, Schoenfeld D, Heine RJ, A1c-Derived Average Glucose Study Group (2008). Translating the A1C assay into estimated average glucose values. Diabetes Care.

[18] Kazemi E, Hosseini S, Bahrampour A, Faghihimani E, Amini M (2014). Predicting of trend of hemoglobin a1c in type 2 diabetes: a longitudinal linear mixed model. Int J Prev Med.

[19] Rose E, Ketchell D, Markova Tsveti (2003). Clinical inquiries. Does daily monitoring of blood glucose predict hemoglobin A1c levels?. J Fam Pract.

[20] Alhassan Z, Budgen D, Alessa A, Alshammari R, Daghstani T, Al moubayed N (2019). Collaborative Denoising Autoencoder for High Glycated Haemoglobin Prediction.

[21] Alhassan Z, Budgen D, Alshammari R, Daghstani T, McGough A, Al moubayed N (2018). Stacked Denoising Autoencoders for Mortality Risk Prediction Using Imbalanced Clinical Data.

[22] Harrell F E, Lee K L, Mark D B (1996). Multivariable prognostic models: issues in developing models, evaluating assumptions and adequacy, and measuring and reducing errors. Stat Med.

[23] Gómez O, Juristo N, Vegas S, editors (2010). Replications types in experimental disciplines.

[24] Lindsay RM, Ehrenberg ASC (1993). The Design of Replicated Studies. The American Statistician.

[25] Kuhn M, Johnson K (2013). Applied Predictive Modeling.

[26] Stone CJ (1986). [Generalized Additive Models]: Comment. Statist Sci.

[27] Austin PC, Steyerberg EW (2012). Interpreting the concordance statistic of a logistic regression model: relation to the variance and odds ratio of a continuous explanatory variable. BMC Med Res Methodol.

[28] Saito T, Rehmsmeier M (2015). The precision-recall plot is more informative than the ROC plot when evaluating binary classifiers on imbalanced datasets. PLoS One.

[29] Batista GEAPA, Prati RC, Monard MC (2004). A study of the behavior of several methods for balancing machine learning training data. SIGKDD Explor. Newsl.

[30] Al Dawish Mohamed Abdulaziz, Robert Asirvatham Alwin, Braham R, Al Hayek Ayman Abdallah, Al Saeed A, Ahmed Rania Ahmed, Al Sabaan Fahad Sulaiman (2016). Diabetes Mellitus in Saudi Arabia: A Review of the Recent Literature. Curr Diabetes Rev.

[31] Centers for Disease Control and Prevention (2020). National Diabetes Statistics Report, 2020. Centers for Disease Control and Prevention.

[32] Baan CA, Ruige JB, Stolk RP, Witteman JC, Dekker JM, Heine RJ, Feskens EJ (1999). Performance of a predictive model to identify undiagnosed diabetes in a health care setting. Diabetes Care.

[33] Griffin SJ, Little PS, Hales CN, Kinmonth AL, Wareham NJ (2000). Diabetes risk score: towards earlier detection of type 2 diabetes in general practice. Diabetes Metab Res Rev.

[34] Williams DRR, Wareham NJ, Brown DC, Byrne CD, Clark PMS, Cox BD, Cox LJ, Day NE, Hales CN, Palmer CR (1995). Undiagnosed glucose intolerance in the community: the Isle of Ely Diabetes Project. Diabet Med.

[35] Kinmonth A, Spiegal N, Woodcock A (1996). Developing a training programme in patient-centred consulting for evaluation in a randomised controlled trial; diabetes care from diagnosis in British primary care. Patient Educ Couns.

[36] Bassiony MM (2009). Smoking in Saudi Arabia. Saudi Med J.

[37] Elhadd TA, Al-Amoudi AA, Alzahrani AS (2007). Epidemiology, clinical and complications profile of diabetes in Saudi Arabia: a review. Ann Saudi Med.

[38] Boffetta P, McLerran D, Chen Y, Inoue M, Sinha R, He J, Gupta PC, Tsugane S, Irie F, Tamakoshi A, Gao Y, Shu X, Wang R, Tsuji I, Kuriyama S, Matsuo K, Satoh H, Chen C, Yuan J, Yoo K, Ahsan H, Pan W, Gu D, Pednekar MS, Sasazuki S, Sairenchi T, Yang G, Xiang Y, Nagai M, Tanaka H, Nishino Y, You S, Koh W, Park SK, Shen C, Thornquist M, Kang D, Rolland B, Feng Z, Zheng W, Potter JD (2011). Body mass index and diabetes in Asia: a cross-sectional pooled analysis of 900,000 individuals in the Asia cohort consortium. PLoS One.

[39] Yoon K, Lee J, Kim J, Cho JH, Choi Y, Ko S, Zimmet P, Son H (2006). Epidemic obesity and type 2 diabetes in Asia. Lancet.

[40] Hu FB (2011). Globalization of diabetes: the role of diet, lifestyle, and genes. Diabetes Care.

[41] Wischmeyer T, Rademacher T (2020). Regulating Artificial Intelligence.

[42] Harerimana G, Kim JW, Yoo H, Jang B (2019). Deep Learning for Electronic Health Records Analytics. IEEE Access.

[43] Abadi M, Chu A, Goodfellow I, McMahan H, Mironov I, Talwar K (2016). Deep learning with differential privacy.

