# Supplementary material for: Predicting Current Glycated Hemoglobin Levels in Adults From Electronic Health Records: Validation of Multiple Logistic Regression Algorithm
Source: JMIR Med Inform. 2020 Jul 3;8(7):e18963. doi: 10.2196/18963 (PMC7367516; doi:10.2196/18963)
Supplement: Multimedia Appendix 1 [file medinform_v8i7e18963_app1.pdf]

Multimedia Appendix 1

Formulae for the calculated variables*:

Non-HDL Cholesterol = Total Cholesterol – HDL Cholesterol

Body Mass Index (BMI) = Weight(kg) / $(\text{Height(m)})^2$

*Reference:
https://www.whittington.nhs.uk/document.ashx?id=10724
https://www.thecalculatorsite.com/articles/health/bmi-formula-for-bmi-calculations.php
