# Supplementary material for: Predicting Current Glycated Hemoglobin Levels in Adults From Electronic Health Records: Validation of Multiple Logistic Regression Algorithm
Source: JMIR Med Inform. 2020 Jul 3;8(7):e18963. doi: 10.2196/18963 (PMC7367516; doi:10.2196/18963)
Supplement: Multimedia Appendix 2 [file medinform_v8i7e18963_app2.pdf]

Multimedia Appendix 2

Lab test and diagnostic codes

Table 5. Lab test codes used by KAIMRC.

Lab Test Code	Description	Units used
L3000002	estimated Glomerular Filtration Rate (eGFR)	mL/min/1.73 m2
L3900229	Random Blood Sugar (Glucose) Level (RBS)	mmol/L
L3000026	Low Density Lipoprotien (LDL)	mmol/L
L3000006	Total Cholesterol (CHOL)	mmol/L
L3000013	High Density Lipoprotien (HDL)	mmol/L

Table 6. ICD10 Hyperglycemia diagnostic codes used by KAIMRC.

Diagnostic Code	Description
E11	Type 2 Diabetes Mellitus
E14	Diabetes Mellitus
E10	Type 1 Diabetes Mellitus
E139	Familial Diabetes Mellitus
R73	Hyperglycemia
O24	Gestational diabetes
