# Supplementary material for: Predicting Current Glycated Hemoglobin Levels in Adults From Electronic Health Records: Validation of Multiple Logistic Regression Algorithm
Source: JMIR Med Inform. 2020 Jul 3;8(7):e18963. doi: 10.2196/18963 (PMC7367516; doi:10.2196/18963)
Supplement: Multimedia Appendix 3 [file medinform_v8i7e18963_app3.pdf]

Multimedia Appendix 3

Units conversion formulae*:

Total Cholesterol (CHOL) and Non-High Density Lipoprotien (non-HDL):
From mmol\L to mg\dL: Multiply by 38.67.
From mg\dL to mmol\L: Multiply by 0.02586.

Random Blood Sugar (Glucose) Level (RBS):
From mmol\L to gm\dL: Multiply by 18.
From gm\dL to mmol\L: Multiply by 0.01129.

*Reference:
https://www.ncbi.nlm.nih.gov/books/NBK33478/
https://www.diabetes.co.uk/blood-sugar-converter.html#
