# Supplementary material for: Predicting Current Glycated Hemoglobin Levels in Adults From Electronic Health Records: Validation of Multiple Logistic Regression Algorithm
Source: JMIR Med Inform. 2020 Jul 3;8(7):e18963. doi: 10.2196/18963 (PMC7367516; doi:10.2196/18963)
Supplement: Multimedia Appendix 4 [file medinform_v8i7e18963_app4.pdf]

Multimedia Appendix 4

PM3 Calculator details

Table 4. PM3 Calculator details for predicting HbA1c level.

Intercept		
	-	-4.5404143
Random Blood Sugar (Glucose) Level (RBS)		
	-	0.010264092 * RBS
	+	5.9135831e-05 * max(RBS - 79.2,0)^3
	-	0.00015189117 * max(RBS - 93.6,0)^3
	+	0.00010465775 * max(RBS - 106.2,0)^3
	-	1.1784309e-05 * max(RBS - 131.4,0)^3
	-	1.181092e-07 * max(RBS - 277.2,0)^3
estimated Glomerular Filtration Rate (eGFR)		
	+	0.040416671* eGFR
	-	5.6881504e-06 * max(eGFR - 15,0)^3
	+	4.98899e-05 * max(eGFR - 74,0)^3
	-	8.7199708e-05 * max(eGFR - 92,0)^3
	+	4.8759935e-05 * max(eGFR - 105.92507,0)^3
	-	5.761977e-06 * max(eGFR - 130,0)^3
Age		
	+	0.074906466 * AGE
	-	7.6062958e-06 * max(Age - 25,0)^3
	-	1.1470552e-05 * max(Age - 44,0)^3
	-	2.2987067e-05 * max(Age - 55,0)^3
	+	9.3130665e-05 * max(Age - 66,0)^3
	-	5.106675e-05 * max(Age - 82,0)^3
Total Cholesterol (CHOL)		
	-	-0.021457758 * CHOL
	-	1.2304102e-06 * max(CHOL - 105.1824,0)^3
	+	5.0953941e-06* max(CHOL - 138.8253,0)^3
	-	5.9498557e-06* max(CHOL - 163.5741,0)^3
	+	2.1424285e-06* max(CHOL - 191.0298,0)^3
	-	5.7556768e-08* max(CHOL - 242.8476,0)^3
Non-High Density Lipoprotien (non-HDL)		
	+	0.034382335 * non_HDL
	-	5.0345293e-06 * max(non_HDL - 72.3129,0)^3
	+	1.4439644e-05 * max(non_HDL - 99.3819,0)^3
	-	1.1919275e-05 * max(non_HDL - 120.2637,0)^3
	+	2.6133174e-06 * max(non_HDL - 146.1726,0)^3
	-	9.915721e-08 * max(non_HDL - 196.8303,0)^3
