# Supplementary material for: Predicting Current Glycated Hemoglobin Levels in Adults From Electronic Health Records: Validation of Multiple Logistic Regression Algorithm
Source: JMIR Med Inform. 2020 Jul 3;8(7):e18963. doi: 10.2196/18963 (PMC7367516; doi:10.2196/18963)
Supplement: Multimedia Appendix 5 [file medinform_v8i7e18963_app5.pdf]

Multimedia Appendix 5

Variables used in the studies

Predictors	Wells et al.	Baan et al.	Griffin et al.	Our study
Age	√	√	√	√
Gender	×	×	√	×
Height	×	×	×	×
Weight	×	×	×	×
Body Mass Index (BMI)	√	√	√	√
Gestational diabetes	×	√	×	×
Prevalence of cardiovascular disease	×	√	×	×
History of diabetes	×	×	√	×
Prescribed antihypertensive medication	×	√	√	×
Prescribed lipid-lowering medication	×	√	√	×
Prescribed steroids medication	×	×	√	×
estimated Glomerular Filtration Rate (eGFR)	√	×	×	√
Random Blood Sugar (Glucose) Level (RBS)	√	×	×	√
Non-High Density Lipoprotien (non-HDL)	√	×	×	√
Total Cholesterol (CHOL)	√	×	×	√
Race	√	×	×	×
Smoking status	√	×	√	×
Physical Activities	×	×	√	×
